# Cardiac arrest, mitral annular disjunction, and mitral valve prolapse: where there is smoke, there is a fire

**DOI:** 10.1093/ehjci/jeae079

**Published:** 2024-03-28

**Authors:** Kristina H Haugaa, Eivind W Aabel

**Affiliations:** Department of Cardiology, ProCardio Center for Innovation, Oslo University Hospital, Rikshospitalet, Sognsvannsveien 20, 0372 Oslo, Norway; Institute of Clinical Medicine, Faculty of Medicine, University of Oslo, Problemveien 11, 0313 Oslo, Norway; Department of Cardiology, ProCardio Center for Innovation, Oslo University Hospital, Rikshospitalet, Sognsvannsveien 20, 0372 Oslo, Norway; Institute of Clinical Medicine, Faculty of Medicine, University of Oslo, Problemveien 11, 0313 Oslo, Norway

**This editorial refers to ‘Mitral annular disjunction in idiopathic ventricular fibrillation patients: just a bystander or a potential cause?’, by L.M. Verheul *et al*., https://doi.org/10.1093/ehjci/jeae054.**

## Editorial comment

The recognition of mitral annular disjunction (MAD) has garnered significant attention in recent years. Initially debated for its clinical significance, MAD seems to be a feature of normal hearts when located in the proximity to the mitral leaflet commissures.^1–3^ However, inferolateral MAD has emerged as a pathological finding and is particularly associated with mitral valve prolapse (MVP) and abnormal left ventricular myocardial mechanics.^1,2^ Other studies have elucidated a compelling link between inferolateral MAD, MVP, and ventricular arrhythmias,^4^ with the phenotypic expression termed arrhythmic MVP (AMVP).^5^ Whether inferolateral MAD could be a harbinger of ventricular arrhythmias without concomitant MVP remains to be elucidated. This is of particular importance due to the difficulty of separating true MAD from pseudo-MAD.^6^ This separation is not always distinct and makes it difficult to compare the presence of inferolateral MAD between studies.

In this issue of EHJ CVI, Verheul *et al*.^7^ assessed the prevalence of MAD and MVP in 185 patients with idiopathic ventricular fibrillation (IVF) undergoing cardiac magnetic resonance (CMR) imaging (mean age 39 years, 40% female). This study expanded on their previous study^8^ showing a higher prevalence of both MAD and MVP in IVF patients compared with healthy matched controls. Their present study now included nine centres in the Netherlands and one centre in London and had more than three times (185 patients in the present study) as many IVF patients. The results were largely consistent, and the increased sample size allowed for more detailed exploration.

MAD at any location was found in 61% (mostly located in the anterior and inferior mitral annulus), consistent with findings in the normal population.^1^ Inferolateral MAD was found in 13% of patients and was associated with MVP (found in 7%). Interestingly, all the MVP patients had inferolateral MAD.

**Figure 1:**
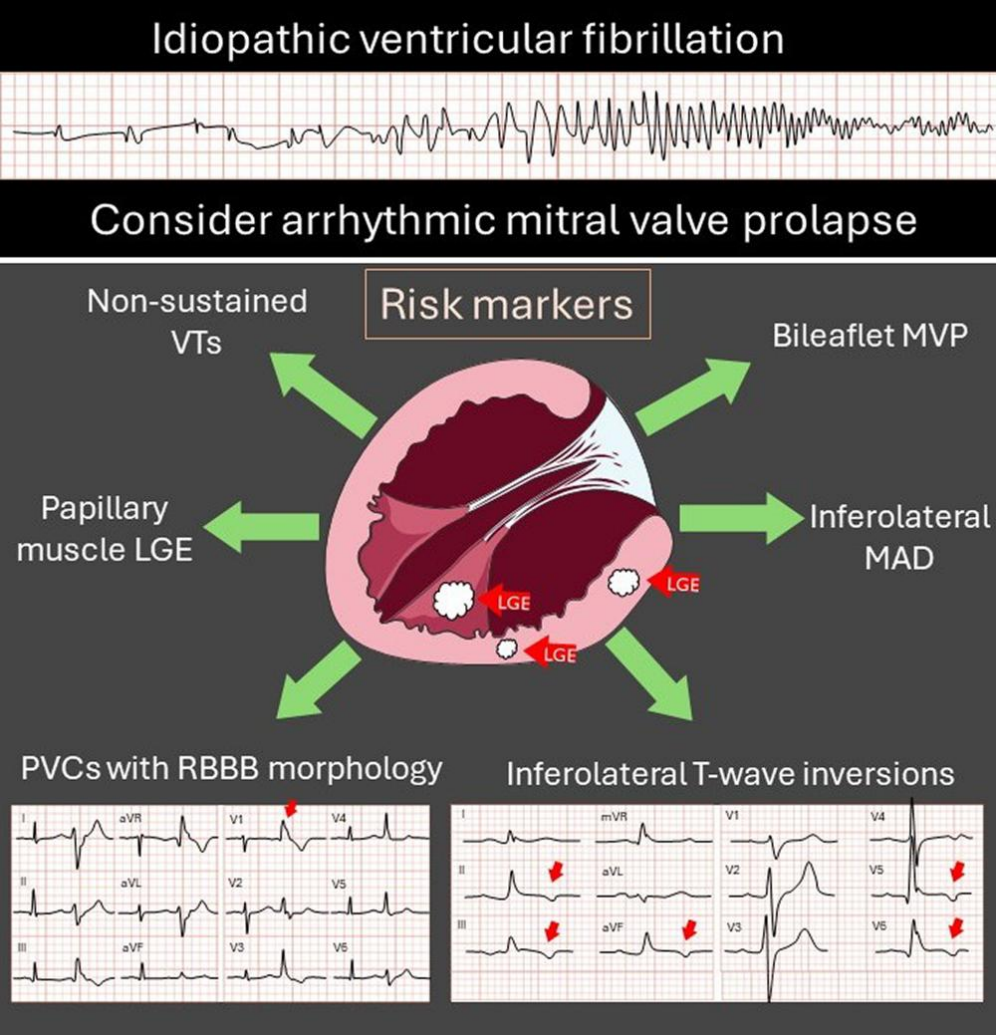
Risk markers and features of arrhythmic mitral valve prolapse. LGE, late gadolinium enhancement; MAD, mitral annular disjunction; MVP, mitral valve prolapse; PVC, premature ventricular complexes; RBBB, right bundle branch block; VT, ventricular tachycardia.

A highly interesting finding was that IVF patients with inferolateral MAD and/or MVP seemed to have a distinct phenotypic arrhythmic expression compared with the other IVF patients. These patients had higher PVC burden, more NSVTs, more T-wave abnormalities in the inferior leads, and more frequent use of antiarrhythmic medication. Together, these features are reminiscent of AMVP (*Figure 1*).

Late gadolinium enhancement (LGE) by CMR imaging has been a cornerstone in risk stratification in AMVP patients.^9^ In the present study,^7^ LGE was found in 13 patients (7%), of which 70% had LGE in the inferior/inferolateral left ventricle. This is the particular location, together with papillary muscle LGE, frequently described in AMVP patients.^9^ However, there was no difference in the presence of LGE between patients with inferolateral MAD and no inferolateral MAD in the present study. Interestingly, the authors found no papillary muscle LGE. However, one may speculate that findings of concomitant cardiac arrest, papillary muscle LGE, and MVP, which are mostly pathognomonic for AMVP, may have excluded these patients from entering the IVF cohort in the first place.

Arrhythmias occurring in AMVP are considered to occur in the myocardium adjacent to the mitral valve apparatus, and they are typically multifocal and complex.^5^ Thus, evaluating PVC and VT morphology and complexity seems crucial when considering an AMVP diagnosis. In the study by Verheul *et al*.,^7^ PVC morphology in patients with inferolateral MAD was mostly multifocal. However, some patients with inferolateral MAD had high PVC burden with a suggested origin from the right or left outflow tracts. Even though PVCs from the outflow tracts have been described in patients with MVP,^10^ it is still a matter of debate whether MVP patients with isolated outflow tract PVCs should be diagnosed with AMVP. Whether these patients have AMVP, or another diagnosis entirely, remains to be proven.

AMVP is associated with a high recurrence rate of life-threatening events in survivors of cardiac arrest, with an incidence rate of 4–8% per year.^11,12^ Verheul *et al*.^7^ also found a high incidence of appropriate ICD therapy during follow-up of 18% over a median follow-up duration of 5 years (approximately 4% per year). However, the incidence rate was similar in IVF patients with inferolateral MAD and no inferolateral MAD.

In summary, is MAD just a bystander or a potential cause in IVF? Well, where there is smoke, there is a fire. We applaud the authors for highlighting the unexplainable cardiac arrests. This important study adds to the evidence that AMVP needs to be considered a potential cause when found in survivors of cardiac arrest. A comprehensive evaluation of cardiac arrest survivors should include a detailed assessment of the mitral apparatus to find inferolateral MAD or MVP. We encourage other centres to review patients with unexplained cardiac arrest to detect possible inferolateral MAD or MVP to add knowledge in the remaining gaps regarding AMVP and IVF and the potential interaction.

## Data Availability

No new data were generated or analysed in support of this research.
